# Editorial: Celebrating 40 Years of the Chilean Society of Pharmacology

**DOI:** 10.3389/fphar.2020.623195

**Published:** 2020-12-17

**Authors:** Gonzalo E. Yévenes, Javier A. Bravo, Guillermo Díaz-Araya, Ramón Sotomayor-Zárate, Jenny L. Fiedler, Miguel Reyes-Parada, Jorge Fuentealba

**Affiliations:** ^1^ Department of Physiology, University of Concepcion, Concepción, Chile; ^2^ Instituto de Química, Facultad de Ciencias, Pontificia Universidad Católica de Valparaíso, Valparaíso, Chile; ^3^ Department of Pharmacological and Toxicological Chemistry, Faculty of Chemical and Pharmaceutical Sciences, University of Chile, Santiago, Chile; ^4^ Centro de Neurobiología y Fisiopatología Integrativa (CENFI), Instituto de Fisiología, Facultad de Ciencias, Universidad de Valparaíso, Valparaíso, Chile; ^5^ Department of Biochemistry and Molecular Biology, Faculty of Chemistry and Pharmaceutical Sciences, Universidad de Chile, Santiago, Chile; ^6^ Centro de Investigación Biomédica y Aplicada (CIBAP), Escuela de Medicina, Facultad de Ciencias Médicas, Universidad de Santiago de Chile, Santiago, Chile; ^7^ Facultad de Ciencias de la Salud, Universidad Autónoma de Chile, Talca, Chile

**Keywords:** pharmacology, addiction, pain, hypertension, receptor

Pharmacology can be defined as the study of the effects of chemical substances on the function of living organisms. Thus, pharmacology is essentially a translational discipline, bridging together biomedical and chemical sciences with the clinical practice. Therefore, the formation and growing of Pharmacological Societies around the world has been, and will continue to be, key in the development of integrated basic, experimental, and clinical sciences.

This Research Topic aimed to celebrate the Chilean Society of Pharmacology (SOFARCHI) 40th anniversary by providing a diverse collection of in-depth reviews and original articles coming from members of our Society and colleagues around the world. SOFARCHI is a non-profit scientific society whose main objective is the promotion of research in pharmacology, from theoretical to experimental, and to clinical points of view. SOFARCHI was founded in 1978 with about 60 members who carried out teaching and research in pharmacology mainly in the central (Santiago and Valparaíso) and in southern zone (Concepción, Valdivia, and Temuco) of Chile. Since 1978, SOFARCHI has held 41 annual congresses and currently has more than 140 members. In addition, SOFARCHI encourages the development and dissemination of pharmacology through promoting research among its members, sponsoring undergraduate and graduate courses, and fostering the creation of teaching books on different pharmacological topics. The Society also actively aids the Institute of Public Health, an agency of the Chilean state, in the technical evaluation of medicines that require sanitary registration. This highlights the commitment of SOFARCHI to promote the proper use of medicines in Chile, making known the main therapeutic and adverse effects of drugs to our fellow citizens. Lastly, the Society has developed a strong outreach program engaging our scientific work to high school students along the country.

Over the last 10 years SOFARCHI has experienced important growth. The annual congress, our main yearly scientific activity, brings together more than 250 attendees, where about 70% of them are undergraduate and graduate students. Thus, SOFARCHI as a young society is always poised to contribute to Chile’s scientific and technological development, but also ready to collaborate with other partners in Latin America, and the world. Therefore, we believe that this research topic brings together many of the main lines of research carried out by Chilean pharmacologists. We are grateful to the Frontiers in Pharmacology Editorial team and many international experts from the field of pharmacology who have been able to support the research topic with their expertise, serving as editors and reviewers.

We are glad that our topic generated an important global impact, reaching more than 50,000 article views and more than 12,000 downloads. The articles published in our research topic include reviews, mini-reviews, perspectives, brief research reports and original research articles. Our topic is grateful to the 240 authors that made their scientific contributions by submitting work of excellence to the Topic.

We would like to first highlight the broad thematic spectrum of the reviews and mini-reviews published in the issue, covering from molecular and cellular to pre-clinical and clinical pharmacology. This group of articles includes in-depth reviews on the contribution of the kappa opioid system to dopamine-related compulsive behaviors (Escobar et al.), on the chemical properties of amphetamine analogs with activity as monoamine oxidase inhibitors (Reyes-Parada et al.), on the contribution and relevance of P_2_X receptors in Alzheimer disease and in the toxicity of soluble Aβ peptide oligomers (Godoy et al.) and on novel strategies and perspectives for the treatment of dental pulp-derived pain (Schuh et al.). This group of contributions also included a detailed clinically-oriented review provided by Castillo et al., which summarizes the clinical experience of Chilean pharmacologists who have worked for more than 10 years with dexmedetomidine, a highly selective α2-adrenergic agonist with sedative and analgesic properties, with minimal respiratory effects. On the other hand, the Topic incorporated self-sustained minireviews summarizing and discussing the relevance of benzofurans as potential chemical scaffolds against Alzheimer disease (Cabrera-Pardo et al.), the effects of vitamin C on cancer cells and its therapeutic potential in cancer (Roa et al.), novel drugs targeting pentameric ligand-gated ion channels as potential novel analgesics (Lara et al.), the neurobiological basis of obsessive compulsive disorder (OCD) with focus on glutamate transporter 3 (EAAT3) (Escobar et al.), recent advances on therapies against non-infectious uveitis (Valenzuela et al.) and the beneficial effects of the mifepristone in metabolic syndromes (Diaz-Castro et al.).

The Topic also achieved to gather a significant number of brief research reports and original research articles of a wide thematic diversity. Aspects of molecular and cellular pharmacology on physiological and pathological states of different systems were covered by original articles offering new findings. At the drug-receptor level, Yarur et al. showed that type-2 β-corticotrophin releasing factor receptor forms a stable heteromeric protein complex with type-1 dopamine receptor, offering a new potential therapeutic target in tissues where both receptors are co-expressed, while San Martin et al., described the functional actions of serotonin type-3 receptor antagonists on glycine receptors associated with pain control. New studies focused on molecular and cellular neuropharmacology were also tackled. In the context of Alzheimer disease, Panes et al., demonstrated that soluble oligomers of Aβ peptide alter key proteins of mitochondrial dynamics and biogenesis in hippocampal neurons, contributing to neuronal dysfunction and death. In terms of potential side-effects of common drugs at the cellular level, Ampuero et al. showed that chronic administration of the fluoxetine reduces dendritic arborization in cortical and limbic areas of the rat brain, suggesting that the permanent SSRI consumption can go beyond the expected antidepressant effects. Furthermore, lines of research focused on cardiovascular and renal pharmacology also contributed to the topic by studying aspects of IFN-β and pro-renin signaling. In this context, Bolivar et al., showed that IFN-β exerts both pro-inflammatory and anti-inflammatory effects on non-stimulated rat cardiac fibroblasts through differential activation of STAT proteins, establishing a new framework for the complex effects of IFN-β on cells of the cardiovascular system, while Reyes-Martinez et al., showed that the pharmacological inhibition of COX-2 in the collecting duct cells might prevent tubular damage after the activation of the intratubular renin–angiotensin system, which may contribute to control renal failure in hypertension and diabetes.

Other original contributions to our topic covered relevant issues with impact on human health, such as chronic pain, cancer, addiction, hypertension, and chronic kidney disease. Regarding neural mechanisms underlying chronic pain, Retamal et al. presented original research showing that central sensitization in rat spinal cord occurs through BDNF release and an increased expression of glial BDNF and pTrkB. In terms of potential novel therapies and strategies against chronic pain, Garrido-Suárez et al. show that mangiferin, a glucosylxanthone broadly distributed in higher plants such as *Mangifera indica L*., has an anti-allodynic effect in a model of chronic post-ischemia pain, while Gonzalez et al., have shown that the antinociceptive efficacy of the common opioid drug, methadone, is potentiated by the co-application of magnesium salts in the spared nerve injury mouse model of chronic pain. In the field of cancer research, two groups of researchers provide new insights. Using biopsies of gastric cancer, Hevia et al. have shown that several types of purinergic receptors, such as P_2_Y_2_R and P_2_X_4_R, are involved in gastric cancer. Interestingly, the authors found a correlation between the expression levels of specific purinergic receptors and the survival rates of patients. In parallel, Lavanderos et al., provide pharmacogenetic evidence as a potential tool to predict adverse drug reactions in Chilean patients diagnosed with testicular cancer. In another study, Robles-Planells et al., demonstrated that the organic extract of *Lithraea caustic*, an endemic stinging plant of central Chile, decreases melanoma tumor growth in a murine model. In addition, other scientific contributions have focused attention on addiction. Velasquez et al., demonstrated that a neonatal programming with estradiol enhances the pharmacological effects produced by morphine in rats, suggesting that early exposure to sex hormones may contribute to opioid addiction in adulthood; on the other hand, Quiroz et al., have shown that a competitive nAChR antagonist (UFR2709) significantly reduced the ethanol consumption of alcohol-preferring rats. Finally, and related to human hypertension, Gonzalez et al., elucidated a biochemical pathway which could underlie the antihypertensive effect of dietary potassium (K^+^) observed in human studies. Interestingly, many of the blood pressure lowering actions of K^+^ were seen in both hypertensive and normotensive rats, supporting a beneficial effect of dietary potassium in health and disease. Similarly, Vio et al., showed that a chronic high potassium diet down regulates key components of intratubular renin-angiotensin system. This down regulation could be a novel mechanism involved in the potassium-induced natriuresis that is beneficial for blood pressure and cardiovascular health. In the same field, Mondaca-Ruff et al., demonstrated a relationship between autophagy, the AT1R/RhoA/Rho Kinase-dependent pathway and Ang II-induced hypertrophy of vascular smooth muscle cells. Last, but not least, Figueroa et al., demonstrated that the pro-inflammatory phase of unilateral ureteral obstruction, a model of Chronic Kidney Disease, involves tubular damage with cortical upregulation of juxtaglomerular renin and medullary (pro)renin receptor downregulation. This scenario suggests a differential iRAS modulation as part of the mechanisms involved in the early stages of chronic kidney disease.

The thematic diversity of our topic also includes articles focused on developmental aspects and on medicinal chemistry. The original research contributed by De La Fuente-Ortega et al., demonstrated that prenatal ethanol exposure affects iron homeostasis of specific brain areas (i.e., PFC and hippocampus), suggesting a critical role of iron homeostasis in maladaptive cognition observed in fetal alcohol spectrum disorder. In addition, Benavides-Rivas et al., described the role of enzymatic glutamate synthesis in the neurulation process using *Xenopus oocytes*. Finally, original research article of Sáez-Briones et al., studied the novel compound 2-Br-4,5-MDMA, a MDMA (3,4-methylenedioxy-methamphetamine, “Ecstasy”) analog brominated at C(2) of the aromatic ring. Interestingly, 2-Br-4,5-MDMA maintains the effects of MDMA as a substrate of the serotonin transporter (SERT). However, the bromination of MDMA modified the expected behavioral responses in rats, showing an absence of the classical MDMA-elicited behavioral responses including hyperlocomotion, enhanced active avoidance conditioning responses and increased social interaction.

In conclusion, we believe that the diversity of articles incorporated in our Research Topic reflects the integrative spirit of our scientific society and the translational character of the pharmacological sciences. In addition, we think that the new findings published show the growing quality of the Chilean science and highlight the rising contribution of our scientists to the global knowledge. We look forward to celebrate many years more of the SOFARCHI.

## Author Contributions

All the authors have equally contributed to the work. The authors reviewed and approved the text for publication.

## Conflict of Interest

The authors declare that the research was conducted in the absence of any commercial or financial relationships that could be construed as a potential conflict of interest.

